# Edible Coating for Fresh-Cut Fruit and Vegetable Preservation: Biomaterials, Functional Ingredients, and Joint Non-Thermal Technology

**DOI:** 10.3390/foods13233937

**Published:** 2024-12-06

**Authors:** Mengjie Ma, Yueyue Liu, Shuaizhong Zhang, Yongkai Yuan

**Affiliations:** 1 College of Food Science and Engineering, Qingdao Agricultural University, Qingdao 266109, China; 2 School of Food Science and Technology, Jiangnan University, Wuxi 214122, China; 3 School of Food Science and Engineering, South China University of Technology, Guangzhou 510641, China; 4 Marine Science Research Institute of Shandong Province, Qingdao 266104, China; 5 Qingdao Special Food Research Institute, Qingdao 266109, China

**Keywords:** biological macromolecules, edible coating, fresh-cut fruits and vegetables, preservation

## Abstract

This paper reviews recent advances in fresh-cut fruit and vegetable preservation from the perspective of biomacromolecule-based edible coating. Biomaterials include proteins, polysaccharides, and their complexes. Compared to a single material, the better preservation effect was presented by complexes. The functional ingredients applied in the edible coating are essential oils/other plant extracts, metals/metal oxides, and organic acids, the purposes of the addition of which are the improvement of antioxidant and antimicrobial activities and/or the mechanical properties of the coating. The application of edible coating with other preservation technologies is an emerging method, mainly including pulsed light, short-wave ultraviolet, modified atmosphere packaging, ozonation, and γ-irradiation. In the future, it is crucial to design coating formulations based on preservation goals and sensory characteristics. The combination of non-thermal preservation technology and edible coating needs to be strengthened in research on food preservation. The application of AI tools for edible coating-based preservation should also be focused on. In conclusion, edible coating-based preservation is promising for the development of fresh-cut fruits and vegetables.

## 1. Introduction

The quality of food depends on dynamic changes in nutrition, sensory quality, and microbiological characteristics throughout the entire processing, storage, and sales process. These changes are the result of the interaction between the food and the environment [1]. The shelf life of the food can be decreased by oxidative reactions, respiration, microbial spoilage, and other factors, posing a potential threat to health [2,3,4]. Therefore, it is necessary to apply appropriate preservation strategies to food.

Owing to the advantage of health and convenience for consumption, fresh-cut fruits and vegetables exhibit a broad prospect. However, different from canning, freeze-drying, and spray-drying, a long product shelf life cannot be achieved by the process of fresh cutting. The high moisture content and large number of cut surfaces generated during the processing of fresh-cut fruits and vegetables can lead to texture damage, browning, microbial pollution, and other issues [5]. Methods used for fresh-cut food preservation have been developed, such as modified atmosphere packaging (MAP), sterile water washing, heat treatment, irradiation, film, and coating [6]. Among them, edible coatings and edible films have attracted great interest in the application of fresh-cut food preservation due to their environmentally friendly and cost-effective characteristics.

The concept of coating or film used for food preservation involves a thin layer composed of edible material that can change its permeability to water vapor, thereby achieving barrier lipid oxidation, inhibiting microbial decay, preventing browning, reducing mass loss, reducing respiratory rate, and controlling enzyme activity. In addition, the natural color and valuable volatile flavor of the food can be kept by using a coating or film [7]. In addition, the addition of natural antimicrobial agents, antioxidants, etc., can also enhance the preservation ability of the edible coatings and films. Although there are studies indicating that they are interchangeable [8], coatings and films are completely different. Films are thin layers wrapped around food materials [9] which are used as a material for packaging. Edible coatings are usually considered liquid forms of edible materials, usually sprayed on the surface of food, or food materials immersed in various edible coatings.

**Figure 1:**
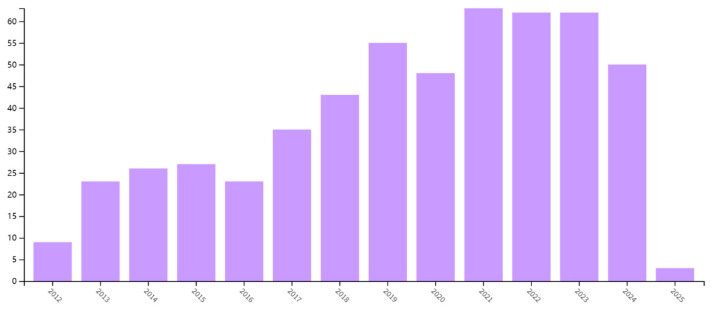
Statistics on edible coatings for fresh-cut fruit and vegetable preservation in Web of Science based on publication year.

For edible coating, extensive research has been conducted on the fabrication of edible coatings with different coating-forming materials and active ingredients applied to coatings. And research on edible coatings involves various types of fruits like apples, kiwifruits, strawberries, and cantaloupe, and vegetables like tomatoes and cucumbers. In addition, many researchers have studied other additives such as antibacterial agents and plasticizers in edible coatings. As shown in Figure 1, research on edible coating for fresh-cut fruit and vegetable preservation has been on an upward trend in the last 15 years, as shown by Web of Science. It emphasizes the increasing importance of this topic of research. However, according to the trend, research reports in the past 5 years seem to have reached a plateau, thus there is an urgent need for relevant reviews to guide the development direction of this field. However, there is currently no comprehensive summary of fresh-cut fruit and vegetable preservation from the perspective of edible coatings.

Therefore, this article aims to summarize the progress made in the study of edible coatings used for the preservation of fresh-cut fruits and vegetables, mainly in the past 5 years. The bio-based materials used for the fabrication of edible coatings are presented. The addition of functional ingredients to edible coatings is introduced. In particular, we discuss research on other preservation technologies that are applied synchronously with edible coatings. The outlook of the fresh-cut preservation of fruits and vegetables presented by edible coatings is given.

## 2. Bio-Based Materials Used for Edible Coatings

Owing to the fact that edible coatings are usually not removed during consumption and cannot have adverse effects on the taste, texture, appearance, etc., of the original food, the materials used for edible coatings must be safe for humans and meet the requirements for appearance and odor. As given in Table 1, various bio-based materials can be applied to the preparation of edible coatings, such as protein, lipid, and polysaccharides, and these components are often mixed in different proportions to improve their mechanical properties. Among them, proteins can serve as an excellent barrier for lipid and gasses and have excellent mechanical strength [2], but protein-based coatings have poor water barrier properties due to their main hydrophilic characteristics [10]. Polysaccharides are also good gas barriers, which means they can effectively reduce lipid oxidation, prevent microbial contamination, and maintain product quality [11,12]. Similarly, owing to hydrophilicity, water vapor barrier function is poor, and has poor mechanical properties, high brittleness, and low elasticity. Lipid-based compounds have strong water barrier properties [2], but they show poor gas barriers [13]. Moreover, lipids typically exhibit micropores, high O_2_ solubility, and diffusivity, leading to unexpected sensory properties, making their application as edible coatings unpopular [14]. Based on the above reasons, a combination of multiple substances is usually applied for the enhancement of the mechanical properties and characteristic functionality of the coating.

### 2.1. Protein

Protein-based materials have strong appeal in edible coatings as they can increase the nutritional value of the coatings [15,16]. Owing to the presence of hydrophilic and hydrophobic regions in the protein, biologically active ingredients can be encapsulated by proteins [17,18]. The substances commonly used as protein bases in edible coatings are gelatin [19], casein [20], whey protein [21], soy protein [22], etc. Gelatin is widely used in coating substrates due to its excellent membrane-forming performance, good biodegradability, low cost, and easy-to-obtain properties [23,24,25]. Meanwhile, it also provides good oxygen and light barrier properties. For example, the results of Li, Zhang, Yang, and Lin showed that the weight loss rate, hardness, pH value, color, and other indicators of fresh-cut apples were significantly better than that of a control without gelatin-based coating [26]. In addition, the experiment conducted by Amiri, Akhavan, Zare, and Radi showed that the gelatin-based coating obtained higher-quality properties than the control group during the storage period of fresh-cut apples, but it did not completely prevent chemical and biochemical reactions [27]. It is precisely due to the high permeability of water vapor and low mechanical property of gelatin-based materials that the best preservation effect cannot be achieved, and the protein functionality of coatings may also be affected adversely by natural enzymes within the foods, such as proteases. Therefore, the issue of natural enzymes hydrolyzing proteins during storage is also worth noting [2].

### 2.2. Polysaccharide

Many natural polysaccharides are excellent materials for the formation of coatings, which is attributed to the role of polar groups like hydroxyl groups [28]. These common polysaccharides include chitosan, alginate, starch, pectin, cellulose, etc. Owing to the ability to form a dense hydrophilic network structure, polysaccharide-based coatings typically serve as an excellent barrier for oxygen, aromatics, and the transport of oil. For the same reasons as proteins, they often serve as poor barriers for water transport as well as poor mechanical properties. Chitosan, as a positively charged polysaccharide, has good biocompatibility, coating-forming properties, and natural antibacterial properties, which are widely used in the fabrication of edible coatings [29]. Chitosan is a unique amino polysaccharide derived from the main component of crustaceans, chitin [30]. And it has the advantages of non-toxicity, biodegradability, and biocompatibility, and has emerging commercial potential in agriculture, food, medicine, and nutrition [31]. Edible chitosan coatings are excellent oxygen permeation barriers and exhibit relatively low barrier properties with water vapor. Moreover, it was found that chitosan exhibits resistance to Gram-negative bacteria and Gram-positive bacteria. The main mechanism of action involves interactions with bacterial cell walls, cell membranes, and cytoplasmic components through electrostatic interactions [32]. For instance, the research results of Ortiz-Duarte, Pérez-Cabrera, Artés-Hernández, and Martínez-Hernández showed that the treatment of coating decreased the respiratory rate, while it did not affect the color, soluble solid content, small molecule sugar, pH value, titratable acidity, citric acid, and malic acid of fresh-cut melon significantly, which means that it can preserve fresh-cut melon [33]. Moreover, Viacava, Cenci, and Ansorena found that all chitosan-coated fresh-cut carrots showed less surface discoloration and better sensory quality [34]. The experiment conducted by Treviño-Garza et al. also obtained similar results, indicating that chitosan-based coating is effective for maintaining the color parameters of fresh-cut cantaloupe and maintaining its overall sensory characteristics [35]. In addition, edible coatings can reduce the survival of aerobic thermophilic microorganisms, molds, and yeast. However, the application of chitosan coatings is limited due to their unexpected mechanical properties, and poor antioxidant activity is also considered a drawback of chitosan coatings. In addition, chitosan derivatives have been found to also serve as coating materials, such as carboxymethyl chitosan [36].

### 2.3. Complexes

**Figure 2:**
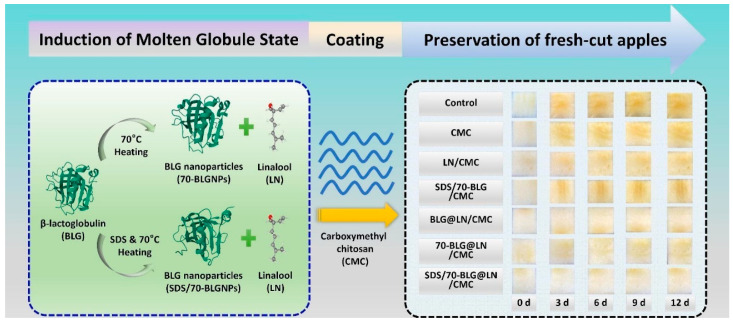
Schematic diagram of carboxymethyl chitosan coating infused with linalool-loaded molten globular β-Lactoglobulin nanoparticles for extended preservation of fresh-cut apples [41].

Owing to the poor gas barrier performance and mechanical properties of lipid-based coatings, lipids are rarely used for independent coating formation. On the contrary, many lipid substances such as glycerol and Tween are widely added to the coating matrix as plasticizers [37,38,39]. As is well known, lipids can enhance the water barrier performance of polymer-based coatings and reduce their water adsorption capacity [40]. They can also serve as carriers of hydrophobic functional ingredients applied to the fabrication of complexes. And precisely due to the poor water barrier properties of polysaccharides and proteins, while poor gas barrier properties are given by lipids, the binding of lipids to polysaccharides and protein matrices is common. In addition, researchers are also attempting to bind polysaccharides and proteins as coating matrix materials. For example, as shown in Figure 2, carboxymethyl chitosan coating infused with linalool-loaded molten globular β-Lactoglobulin nanoparticles could be used to extend the preservation of fresh-cut apples [41]. The binding of gelatin and rice starch as a coating substrate was utilized by Bari and Giannouli, who found that the incorporation of rice starch to the coating of gelatin maintained breaking force and hardness while accelerating the enhancement of total color differences and weight loss [42]. Although the desired effect was not achieved, it also indicated that the coating made of gelatin and rice starch may be useful. In addition, Guo et al. also used the binding of proteins and polysaccharides as a coating matrix, and the authors evaluated the antimicrobial effect of the coating composed of whey protein isolate and pectin [43]. Owing to the adverse characteristics exhibited by a single coating substrate, researchers can conduct combined experiments based on the characteristics of different coating substrates, and considering the characteristics of fruits and vegetables, to find coating substrate materials with expected preservation and mechanical properties.

**Table 1:** Recent bio-based materials used for edible coatings.

Bio-Based Materials		Representative Example		
Classification	Main Type	Application Method of Coating	Effect on Fresh-Cut Fruits and Vegetables	Reference
Protein	Gelatin, casein, whey protein, soy protein, etc.	Soaking followed by drying	Gelatin-based coating obtained higher-quality properties than the control group during the storage period of fresh-cut apples	[27]
Polysaccharide	Chitosan, alginate, starch, pectin, cellulose, etc.	Soaking followed by drying	Chitosan-coated fresh-cut carrots showed less surface discoloration and better sensory quality	[34]
Complexes	Lipids/polysaccharides, lipids/protein, polysaccharides/protein	Soaking followed by drying	Whey protein isolate/high-methoxyl pectin-based carvacrol emulsions effectively delayed the browning process and reduced variation in chemical quality compared to other fresh-cut apples	[43]

## 3. Functional Ingredients Applied in Edible Coatings

The addition of functional ingredients can be divided into two categories based on their effects. One type is used to enhance the mechanical properties of the coating. The other category is used to enhance the preservation activity of coatings by promoting specific functions, such as antioxidant and antimicrobial activities that delay the deterioration, rancidity, or oxidation discoloration caused by free radicals. In addition, if the coating contains sweeteners, spices, and seasonings, it can also improve the sensory characteristics of the coated product [44,45,46]. As shown in Table 2, three types of substances are discussed as follows: essential oils and other plant extracts, metals and metal oxides, and organic acids.

### 3.1. Essential Oils and Other Plant Extracts

Essential oils are widely used to maintain food quality due to their antimicrobial and antioxidant activities and have received increasing attention. The ability of various essential oils to act as antioxidants, antimicrobial agents, and eco-friendly food preservatives has been reported by previous studies [47,48,49,50]. Various essential oils have been applied to edible coatings, such as cinnamon essential oil [51] and *Mentha pulegium* essential oil [52]. Specifically, Sarengaowa et al. studied the antibacterial effect on fresh-cut potatoes using chitosan edible coating containing cinnamon oil, and the results showed that 0.2% cinnamon oil-loaded chitosan emulsion can effectively preserve fresh-cut potatoes, which is attributed to the decrease in the total number of colonies like *Listeria monocytogenes* [51]. However, it was also found that essential oil components may lower the quality of coated products, such as color, hardness, taste, and odor. Galus et al. reported the effect of lemongrass essential oil on the preservation of fresh-cut pears, and the authors found that the presence of essential oils had a significant effect on the reduction in water vapor permeability, carbon dioxide, and oxygen [53]. Lemongrass essential oil can be applied to the fabrication of edible coatings successfully for whey protein isolates. However, for samples coated with formulations containing lemon oil, a decrease in hardness was observed. And it also showed that incorporating essential oils into the protein matrix reduced the acceptability of fresh-cut pears. Thus, when adding essential oils to edible coatings, special attention should be paid to whether they will have adverse sensory effects. Meanwhile, attention should be paid to whether there is external interference that prevents essential oils from exerting their effective effects, as well as the degradation and loss of essential oils.

In addition to essential oils, other plant extracts may also exhibit significant antioxidant and antimicrobial activity, and inhibit the lipid oxidation of foods. At present, various plant extracts have been tested for their antimicrobial activity as additives in paint formulations, such as citrus extract [10], tomato oil extract [54], ginseng extract [55], and *Sonchus oleraceus* L. extract [56]. Plant extracts isolated from agricultural products or traditional Chinese medicine residues have received widespread attention, and their effective utilization can improve resource utilization while avoiding waste and maximizing waste value, such as saffron extract [57], banana peel extract [58], and apple pomace extract [59]. Hashemi and Jafarpour added saffron petal extract to edible coatings, and the results showed that saffron petal extract significantly improved the transparency and water content, reduced the water vapor permeability of the coating, reducing the spoilage of cucumber slices, and maintained the quality characteristics of cucumber slices [57]. Hammad, Elsayed, and Elkashef used concentrated whey protein and apple pomace extract as the main raw materials to study the preservation influence of the coating on fresh-cut apples [59]. The authors found that apple pomace extract contains 15 phenolic compounds. The experiment showed that apple pomace extract had significant antioxidant activity and reduced weight loss, and after storage for some time, the brightness of freshly cut apple slices was higher and the browning was lower. Meanwhile, the authors also found that the addition of apple pomace extract had a certain antibacterial effect and had little impact on the sensory evaluation of apple slices. However, there are studies suggesting that, similarly to essential oils, the addition of natural extracts to edible coatings may produce bitterness, astringency, or odor, thereby damaging the acceptability of the product [60,61]. Therefore, sensory evaluation in this area of study may be necessary.

### 3.2. Metals and Metal Oxides

Metals and metal oxides are also added to edible coatings, among which, zinc oxide (ZnO) nanoparticles, a generally recognized safe metal oxide, have been widely applied in the field of food packaging. It has the advantages of being relatively inexpensive, odorless, and easy to synthesize [6]. Emamifar and Bavaisi fabricated a biological sodium alginate coating containing nano ZnO that can increase the moisture resistance of the coatings significantly [62]. After storage, coated fruits showed lower weight loss than that of uncoated fruits. In addition, compared to crude chitosan solution and chitosan nanoparticles containing the metal ions Fe^2+^, Cu^2+^, Mn^2+^, and Zn^2+^, a higher antimicrobial activity was presented by chitosan nanoparticles loaded with Ag^+^, which is possibly attributed to their ability to bind microbial nucleic acid, proteins, and enzymes [63]. Incorporating Ag nanoparticles (nanocomposites) into the chitosan matrix can also improve the mechanical characteristics, water vapor barrier, and antimicrobial property of coating composed of chitosan [33].

In addition to antibacterial properties, metals and metal oxides can also enhance the mechanical properties of the coating. Calcium ions are commonly used as cross-linking agents in alginate, pectin, and whey protein. Applying a cross-linking agent can form a denser and corrosion-resistant layer after the deposition of coating solution on the food surface, and the thermal stability, chemical resistance, and mechanical strength can be enhanced [64,65]. For example, Amiri, Akhavan, Zare, and Radi used calcium treatment to maintain or improve the hardness and brittleness of coatings [27].

**Table 2:** Functional ingredients applied in edible coatings.

Classification	Main Type	Function	Reference
Essential oils and other plant extracts	Cinnamon essential oil, lemongrass essential oil, saffron extract, apple pomace extract, etc.	Antibacterial activity; Antioxidant activity	[51,53,57,59]
Metals and metal oxides	Zinc oxide nanoparticles	Antibacterial activity	[62]
	Ag nanoparticles	Antibacterial activity; Enhance the mechanical properties of the coating	[33,63]
	Calcium ions	Enhance the mechanical properties of the coating	[27]
Organic acids	Citric acid, ascorbic acid, tannic acid, etc.	Antibacterial activity; Antioxidant activity; Cross-linking agent	[27,66,67,68,69,70]

### 3.3. Organic Acids

Organic acids are commonly used as antimicrobial agents and antioxidants. As a chelating agent for isoascorbic acid and its neutral salts, citric acid can provide an acidic environment that is beneficial to browning resistance and sterilization, which is done to preserve fresh-cut products. For example, Amiri, Akhavan, Zare, and Radi used citric acid and ascorbic acid as substitutes for sulfite in the impregnation treatment of fresh-cut fruits to prevent enzymatic browning after peeling and/or cutting, achieving ideal results [27]. Nascimento, Stamford et al. also added citric acid to the coating matrix, and the results showed that the parameters of the coated fresh-cut guava quality were maintained during storage while preserving its sensory characteristics [66]. In addition, some organic acids can also act as cross-linking agents, such as citric acid cross-linked starch, citric acid cross-linked cellulose derivatives, tannic acid cross-linked gelatin, and tannic acid cross-linked chitosan [67,68,69,70].

## 4. Other Preservation Technologies Combined with Edible Coatings

Research on other preservation technologies that are applied with edible coatings synchronously is an emerging direction for better fresh-cut food preservation. These preservation technologies mainly include pulsed light, short-wave ultraviolet, MAP, ozonation, and γ-irradiation (Table 3). Researchers treat fresh-cut products individually or collectively, store them under the same conditions, and conduct quality and corresponding sensory evaluations during and after storage.

### 4.1. Pulsed Light

Pulsed light treatment is a promising decontamination technology that uses intense, broad-spectrum, short-duration pulses from ultraviolet to near-infrared light to inactivate microorganisms [71]. It has been approved for food surface decontamination, and the efficient treatment of pulsed light in the microbial inactivation of fresh-cut fruits has been reported [72]. The Food and Drug Administration established a maximum cumulative dose of 12 J/cm^2^ in 1996. The application of edible coating can maintain the product’s texture and quality, while repeated pulsed light helps to reduce microbial counts. The combination of the two may achieve better preservation effects by their final synergistic effect.

Recently, Koh, Noranizan, Karim, and Nur Hanani used sodium alginate coating and repeated pulsed light to conduct experiments on fresh-cut Hami melons, and the authors found that the combination of them was more effective in maintaining the total aroma substance concentration and low lactate concentration in fresh-cut Hami melons than the individual treatment after the storage [73]. Importantly, the sensory quality of fresh-cut Hami melons was maintained effectively by the application of alginate coating and repeated pulsed light. In addition, alginate coating treatment was more effective than repeated pulsed light treatment in reducing the changes in organic acids during storage of fresh-cut Hami melons and showed a greater contribution to total aromatic compound concentration. Pirozzi et al. investigated the effect of pulsed light and edible coating on the preservation of tomatoes, and the results exhibited that the joint application of two preservation methods significantly improved the physicochemical properties of tomatoes compared to an untreated group and individual treatments (edible coating or pulsed light alone) [74]. During the entire storage period, there is a decrease in thermophilic aerobic bacteria, yeast, and mold, while minimizing changes in physicochemical properties.

The impact of pulsed light treatment on the physicochemical characterization of edible coating has not been clarified fully. A positive effect is possible, as the UV component of pulsed light may cause a cross-linking reaction within the coating; thereby, its mechanical properties can be improved [75]. Meanwhile, if pulsed light is treated after the deposition of the coating, and the surface of the food is particularly sensitive to light, the final UV shielding effect may be beneficial for food preservation. However, the combination of pulsed light and edible coating brings potential obstacles to widespread industrial applications due to the complexity of the process and the high cost of investment. It should also be noted that the maximum cumulative dose of pulsed light-treated food is 12 J/cm^2^, presented by the Food and Drug Administration, and attention should be paid to the intensity and frequency of repeated pulsed light action.

### 4.2. Modified Atmosphere Packaging

The technology of MAP has been commercially used for food preservation [76]. MAP applications can reduce the respiratory rate, microbial growth, and browning, thus maintaining the quality of fruits and vegetables [77,78,79]. This technology meets the demands of consumers for a healthy and fast-paced lifestyle [80], and it has been widely used to maintain the quality of fresh-cut products. MAP can occur in two ways, one is active MAP, by transporting specific product gasses or gas mixtures into packaging, and the other is passive MAP, which spontaneously transports gasses through the respiration of plants in packaging. However, a single MAP treatment is limited for the preservation of fresh-cut agricultural products and a better result can be achieved when applied with other preservation technologies simultaneously [80].

For example, Karagöz and Demirdöven investigated the changes in polyphenol oxidase activity, antioxidant properties, and microbial quality using a combination of chitosan and MAP technology for the preservation of stevia-coated instant apple slices, and the results showed that the polyphenol oxidase activity, respiratory rate, and pH value of the coated group were lower than those of control group, while the antioxidant capacity and phenolic content were higher than those of the control sample [81]. Moreover, the application of chitosan coating and MAP resisted aerobic mold effectively. Olawuyi, Park, Lee, and Lee reported the influence of chitosan and nitrogen/argon MAP on the preservation of fresh-cut cucumber, and the authors found that the chitosan coating maintained the fresh-cut cucumbers’ quality to a certain extent, but the effect of using chitosan coating alone may not be sufficient for long-term storage [82]. Overall, the joint application of chitosan coating and argon-protected MAP can better preserve the quality of fresh-cut cucumbers and extend their shelf life, with a storage life of twelve days. Therefore, it can be inferred that the joint application of chitosan treatment and argon-based MAP has potential applications in the food industry to maintain the overall quality of fresh-cut cucumbers. Liao et al. studied the influence of the joint application of edible coatings and MAP on the quality of fresh-cut pineapple during storage and its microbiological characteristics, and the authors found that the efficiency of joint application exceeded that of the edible coating or MAP alone [80]. Freshly cut pineapples treated with edible coating+MAP exhibited an excellent appearance, with lower relative conductivity and soluble solids compared to other treatment groups, as well as higher titratable acids, ascorbic acid, total phenolic content, and hardness. At the end of storage, the edible coating+MAP group showed the lowest levels of peroxidase activity, malondialdehyde content, and polyphenol oxidase activity. The results showed that edible coating+MAP treatment can more effectively maintain the quality of fresh-cut pineapple during storage, extending its shelf life.

### 4.3. Short-Wave Ultraviolet

As a non-thermal sterilization technology, short-wave ultraviolet irradiation (UV-C) with 200–280 nm can kill pathogenic bacteria effectively, and has been recognized as a safe method of food processing by the Food and Drug Administration [83]. This technology has been widely applied in various fresh-cut foods to inhibit unexpected flavors, inactivate microbial growth, reduce softening, inhibit browning, delay ripening, and maintain food quality during storage [84,85,86,87,88]. The joint application of short-wave ultraviolet and edible coating is expected to ensure the safety of fresh-cut fruits and vegetables that cannot be heated. The results of Zambrano-Zaragoza et al. exhibited that the joint application of 4.5 kJ/m^2^ short-wave ultraviolet and coating decreased the quality loss of fresh-cut cucumbers, which was related to the decrease in initial microbial load by the treatment of short-wave ultraviolet and the preservation effect of the coating, reducing ROS and thus reducing oxidative reactions (enzymatic and non-enzymatic) [89].

### 4.4. Ozonation

Ozonation is a widely used technology in the food industry, with an efficiency of 1.5 times that of chlorine and no hazardous by-products produced after decomposition [90,91]. Compared to chlorine, ozone can inhibit a large number of microorganisms. Specifically, ozone can inhibit bacteria, molds, yeast, parasites, and viruses [92]. Maherani, Harich, Salmieri, and Lacroix reported the influence of the joint application of ozone and coating on fresh-cut green peppers, and the results showed a significant reduction in all bacteria studied [93]. The authors found that the treatment had no effect on the color parameters of green peppers, and the use of ozone oxidation and coating alone or in combination had no negative impact on the chlorophyll content of peppers. Compared to the control sample, the evaluation of the sensory properties of samples treated with different treatments showed no significant difference, indicating the possibility of further commercialization of the combination of coating treatment and ozone treatment.

### 4.5. γ-Irradiation

γ-irradiation is an effective sterilization method used for many foods [94], is highly penetrating, and can propagate over long distances in the air [95]. It can also penetrate target foods to various depths, and it has high efficiency in killing microorganisms by breaking the covalent bonds between bacterial DNA and viruses [96]. Ben-Fadhel et al. evaluated the preservation of fresh-cut carrot slices treated with calcium caseinate coating and γ-irradiation, and it was found that the irradiation and calcium caseinate coating effectively maintained the mechanical properties of the coating [10]. Compared with a single treatment, the joint application of coating and γ-irradiation showed a synergistic potential and a higher efficiency to extend the shelf life of carrots and maintain their quality throughout storage.

**Table 3:** Examples of other preservation technologies combined with edible coatings.

Technology	Joint Application Example	Performance Parameters	Reference
Pulsed light	The combination of sodium alginate coating and repeated pulsed light has a better preservation effect on fresh-cut Hami melons	A fluence of 0.9 J/cm^2^ was applied every 48 h, and the total cumulative pulsed light fluence was 11.7 J/cm^2^	[73]
Modified atmosphere packaging	The combination of chitosan coating and modified atmosphere packaging has a better preservation effect on fresh-cut apples	Passive modified atmosphere packaging +1 ± 2 °C (at the beginning gas composition: 21% O_2_, 0.03% CO_2_ and other gasses)	[81]
Short-wave ultraviolet	The combination of lemon essential oil alginate-pectin nanoparticles and short-wave ultraviolet has a better preservation effect on fresh-cut cucumber	Short-wave ultraviolet treatment was carried out in a chamber with aluminum-coated walls using a 15 W UVP lamp model XX-15S at 254 nm	[89]
Ozonation	The combination of coating and ozonation has a better preservation effect on ready-to-eat frozen pre-cut green peppers	Ozonation was carried out at 10 ppm for 5 min of ozone exposure at a flow rate of approximately 15 standard cubic feet per hour	[93]
γ-irradiation	The combination of calcium caseinate coating and γ-irradiation has a better preservation effect on fresh-cut carrot slices	γ-irradiation was carried out at a dose rate of 9.622 kGy/h (+/−2.3%) in a cobalt-60 Underwater Calibrator UC-15A (energy level: 1.25 MeV)	[10]

**Figure 3:**
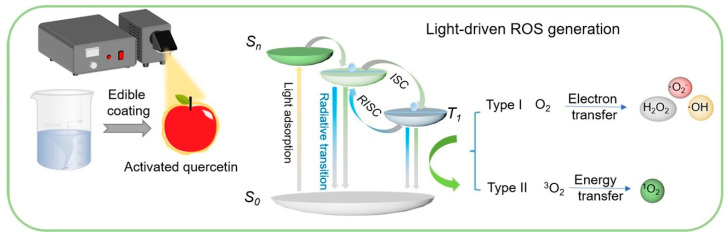
Illustration of the quercetin edible coating with photodynamic bacteria inactivation [98].

Other innovative joint approaches have also been proposed, such as slightly acidic electrolyzed water [97] and photodynamic bacteria inactivation (Figure 3) [98]. At present, there is relatively little research on the joint application of edible coatings and other technologies for preservation, and some studies only evaluate the effectiveness of combination treatments for individual indicators. Based on the results obtained, combination treatments may achieve better preservation effects, and their specific effects and commercialization may require more research results. This requires more researchers to try to comprehensively evaluate preservation effects, to find better preservation strategies for fresh-cut products, and to promote the further development of the fresh-cut-product market.

Based on market research, further discussions were held on the costs of different technologies: (1) Pulsed light systems can be expensive initially, typically ranging from USD 5000 to USD 50,000 depending on size and capability. And its operating costs are generally low due to energy efficiency, and maintenance primarily involves periodic bulb replacement, adding minimal additional costs. (2) The initial investment for MAP machinery is moderate to high, ranging from USD 10,000 to USD 100,000 for larger setups. For packaging cost, the cost of bulk production is around USD 0.05 to USD 0.15 per unit, depending on the specific gas blend and materials used. (3) Portable UV-C units range from USD 1000 to USD 5000, while industrial units are priced between USD 10,000 and USD 30,000. UVC bulbs require replacement every few thousand hours and cost around USD 20–USD 50 each, with low ongoing energy costs. (4) Ozone generators vary in price from USD 500 for smaller units to USD 20,000 for industrial systems. Operating costs primarily come from electricity use, approximately USD 0.01 to USD 0.05 per hour, with some costs for maintenance and occasional generator repair or filter replacements. (5) γ-irradiation is typically performed using specialized facilities, so costs are around USD 0.50 to USD 2 per kilogram depending on dosage and volume.

## 5. Comprehensive Comparison

This section provides a comparative discussion on the above bio-based materials, functional ingredients, and joint non-thermal technology, considering their advantages and disadvantages, specific preservation goals, sensory impacts, performance, and economic value.

As discussed in Section 2, proteins, polysaccharides, and lipids have their advantages and disadvantages in edible coatings, complexes of which can improve the overall functionality of the coating by balancing gas and water barrier properties. Combining materials and complexes can partially address their weaknesses, but may increase production complexity. For preservation goals, bio-based materials aim to maintain the texture, appearance, and shelf life of fresh-cut produce without negatively impacting taste or safety. When well-formulated, biomaterial coatings minimally alter food flavor or texture. Biomaterial coatings are relatively cost-effective and environmentally friendly, aligning well with consumer demand for natural preservation methods. Their efficacy, however, depends on the specific combination of materials and additives used to achieve desired preservation outcomes.

As discussed in Section 3, functional ingredients provide antimicrobial and antioxidant effects, enhancing the preservation capabilities of the coating. For preservation goals, functional ingredients extend shelf life by reducing microbial growth, oxidation, and browning, critical for retaining freshness in fresh-cut products. However, essential oils can impart strong flavors or odors that may be undesirable. Therefore, careful selection and dosage are necessary to avoid negatively affecting sensory qualities. Organic acids must be balanced to avoid sensory issues. While functional ingredients can boost preservation performance, the cost can vary significantly depending on the type and concentration used.

As discussed in Section 4, these technologies offer effective microbial control, delay ripening, and reduce spoilage without directly altering the product’s composition. For instance, UV-C and pulsed light effectively inactivate microorganisms, MAP regulates respiratory activity in packaged produce, and γ-irradiation provides deep penetration for pathogen control. However, the equipment and operational costs can be high. Additionally, some methods may not be suitable for small-scale applications due to their setup and maintenance requirements. For high-value products or large-scale operations, these technologies are economically justified, but may not be viable for small businesses due to the initial and ongoing costs. These technologies are often combined with edible coatings to maximize preservation benefits. Most non-thermal technologies have minimal impact on taste and appearance. However, some, like UV-C and pulsed light, may require adjustments in intensity to avoid affecting sensitive produce.

Bio-based materials are cost-effective and sustainable but may require combinations to achieve desired properties. Functional ingredients add specific preservative functions but must be balanced to avoid altering sensory qualities. Advanced technologies provide strong microbial control and preservation but are generally more expensive and more suitable for large-scale operations. Each category has unique strengths and weaknesses, and the choice depends on the specific preservation goals, desired sensory outcomes, and available budget. Integrating edible coatings with non-thermal technologies like MAP or UV-C may offer the most comprehensive solution by balancing performance with economic feasibility.

## 6. Summary and Outlook

In summary, the preservation technology of edible coatings for fresh-cut fruits and vegetables has made rapid development in the past period. Owing to its environmentally friendly and convenient use characteristics, it has shown good practicality in the preservation of fresh-cut fruits and vegetables. We have reason to believe that with the continuous deepening of research, more and more novel raw materials for coating substrates and additives will be discovered and reported. Similarly to other preservation methods, edible coatings have shortcomings, such as insufficient preservation effects and significant sensory impacts on fruits and vegetables. How to mix coating substrate materials based on the characteristics of different coatings substrates and add corresponding substances based on demand to enhance the mechanical properties, preservation, and sensory effect of the coatings is undoubtedly a key development direction in the future. Compared to the development of edible coatings, the attempt to find a combination of edible coatings and another preservation technology is still in a relatively small area of research. If a suitable combination of edible coatings and another preservation technology can be found, it will be of great significance for the development of fresh-cut fruits and vegetables. In addition, AI tools are expected to assist in the efficient and intelligent development of edible coating-based preservation. For example, a full factorial experimental design and artificial neural network model were deployed to predict and optimize the adhesion characteristics of nanoemulsion coatings sprayed onto plantain epicarps [99].

## Data Availability

No new data were created or analyzed in this study. Data sharing is not applicable to this article.
